# Risk of Infections Secondary to the Use of Targeted Therapies in Hematological Malignancies

**DOI:** 10.3390/life13061272

**Published:** 2023-05-28

**Authors:** Mihaela Andreescu

**Affiliations:** 1 Department of Clinical Sciences, Hematology, Faculty of Medicine, Titu Maiorescu University of Bucharest, 040051 Bucharest, Romania; tevetmihaela@gmail.com; 2 Department of Hematology, Colentina Clinical Hospital, 020125 Bucharest, Romania

**Keywords:** secondary immunodeficiency, immunosuppression, mucosal barrier injury, prophylaxis, chemotherapy

## Abstract

Concurrent infections in hematological malignancies (HM) are major contributors to adverse clinical outcomes, including prolonged hospitalization and reduced life expectancy. Individuals diagnosed with HM are particularly susceptible to infectious pathogens due to immunosuppression, which can either be inherent to the hematological disorder or induced by specific therapeutic strategies. Over the years, the treatment paradigm for HM has witnessed a tremendous shift, from broad-spectrum treatment approaches to more specific targeted therapies. At present, the therapeutic landscape of HM is constantly evolving due to the advent of novel targeted therapies and the enhanced utilization of these agents for treatment purposes. By initiating unique molecular pathways, these agents hinder the proliferation of malignant cells, consequently affecting innate and adaptive immunity, which increases the risk of infectious complications. Due to the complexity of novel targeted therapies and their associated risks of infection, it often becomes a daunting task for physicians to maintain updated knowledge in their clinical practice. The situation is further aggravated by the fact that most of the initial clinical trials on targeted therapies provide inadequate information to determine the associated risk of infection. In such a scenario, a cumulative body of evidence is paramount in guiding clinicians regarding the infectious complications that can arise following targeted therapies. In this review, I summarize the recent knowledge on infectious complications arising in the context of targeted therapies for HM.

## 1. Introduction

Infections remain a significant concern in patients receiving targeted therapies for hematological malignancies (HM) [1]. Patients with HM are inherently susceptible to infectious pathogens due to their impaired immune responses, either as a direct result of their underlying hematological condition or as a consequence of specific therapeutic interventions aimed at targeting the malignancy [2]. Over the years, the treatment paradigm for hematological malignancies has witnessed a tremendous shift, from broad-spectrum treatment approaches to more specific targeted therapies that modify one or more cellular pathways [3]. At present, targeted therapies remain at the forefront of ongoing research in hematological malignancies and are constantly reshaping the therapeutic landscape with novel therapeutic agents [4,5]. Initially, it was believed that the inception of these novel agents would minimize the infectious complication rate post-therapy. However, several unpredictable infectious sequelae have emerged with the use of some of the targeted therapies. Although targeted therapeutic agents demonstrate a narrow spectrum of toxicity, primarily due to their specific signaling pathways, they have the potential to cause downstream path inhibition, which can alter the immune system [6]. Consequently, prolonged immunosuppression in such patients exposes them to opportunistic pathogens. A broad array of pathogenic agents, such as fungi, protozoa, and viruses, have been identified in HM patients undergoing some targeted therapies [7,8].

Due to the constantly changing therapeutic landscape of HM and the advent of novel targeted therapies, it has become a daunting task for clinicians to keep track of the potential infectious complications that can arise after treatment. Generally, it has been noted that most infectious disease physicians often exhibit a lack of comprehensive understanding regarding the fundamental physiological processes or undesirable effects associated with the use of specific targeted therapies [9]. Therefore, it is critical for them to develop a deeper understanding and maintain updated knowledge regarding the unique risks that are associated with targeted therapies. However, most clinical studies reporting infectious complications in hematological malignancies provide incomplete data that rarely permit a structured presentation [10]. In such a scenario, a cumulative body of evidence is paramount in guiding clinicians regarding the infectious complications that can arise following targeted therapies. In this review, I summarize recent innovations, from an infectious complication perspective, regarding targeted therapies in hematological malignancies. I cover targeted therapies that are frequently utilized in HM treatment, including different monoclonal antibodies, bispecific T-cell engagers (BiTE), Bruton’s tyrosine kinase (BTK) inhibitors, and Janus-associated kinase (JAK) inhibitors, as well as the B-cell lymphoma 2 (BCL-2) inhibitor (Table 1).

## 2. Monoclonal Antibodies

Due to their higher specificity and low adverse reactions, therapeutic monoclonal antibodies have emerged as the predominant drugs in the development phase [23]. As of 2022, eighty monoclonal antibodies have been approved by the Food and Drug Administration (FDA) for use [24,25]. To facilitate the distinction between various monoclonal antibodies, an international nomenclature has been proposed, using specific suffixes corresponding to their biological origins, such as murine, chimeric, humanized, or human antibodies [26]. Murine antibodies (suffix “-omab”) were the first to be formed from rodent sequences. However, since then, the utilization of more sophisticated engineering technologies has led to more specified antibodies, such as chimeric (suffix “-ximab”) from the combination of murine Fab and human Fc [27]. Humanized antibodies are denoted by the suffix “-zumab” and are primarily formed by human sequences but have complementarity-determining regions (CDRs) of murine origin. Human antibodies (suffix “-umab”) demonstrate weaker immunogenic properties and are developed purely from human sequences [28].

### 2.1. Anti-CD20 Monoclonal Antibodies

The inception of anti-CD20 mAbs was hailed as a groundbreaking event [29,30]. Anti-CD20 mAbs are a family of treatment therapies targeted at CD20-positive B-cell malignancies and other orphan autoimmune diseases [31,32]. CD20 is a B-cell-specific membrane protein that is expressed on normal and malignant B-cells but not on B-cell precursors or plasma cells; therefore, anti-CD20 mAbs do not cause immediate impairments in immunoglobulin production [33]. Repeated doses of some anti-CD20 antibodies have been associated with hypogammaglobulinemia and the late onset of neutropenia (LON) [34]. The underlying mechanism of immunosuppression by anti-CD20 mAbs includes long-lasting B-cell depletion either by apoptosis or cell-mediated cytotoxicity, which consequently results in alterations in humoral immune responses [35]. Following anti-CD20 monoclonal antibody treatment, a decrease in the B-cell population may persist for at least 6 to 9 months [9]. Further anti-CD20 antibodies impart various downstream effects by influencing the function of B- and T-cells regarding antigen presentation and cytokine production [36].

Rituximab was the first anti-CD20 monoclonal antibody that was approved for the treatment of lymphoid malignancies [37]. Rituximab is of chimeric origin and binds to the CD20 antigen present in all peripheral B-cells. Rituximab is indicated for relapsed/refractory, follicular B-cell non-Hodgkin’s lymphoma (NHL), newly diagnosed or previously treated CLL, microscopic polyangiitis (MP), rheumatoid arthritis, and systemic lupus erythematosus (SLE) [38,39,40,41,42,43]. Late onset of neutropenia has emerged as a frequently reported consequence of rituximab, either as a monotherapy or combination therapy [44]. Shimony et al. shared findings from 330 study participants with lymphoproliferative neoplasms who were categorized into rituximab (*n* = 283) and obinutuzumab (*n* = 47) treatment groups [45]. Late onset of neutropenia was observed in 23% of patients who were present in the rituximab arm of the study [45]. Similar results were shared by Tesfa et al., who investigated 169 evaluable consecutive rituximab-treated NHL patients [46]. Fifteen patients (9%) in the treatment group developed late-onset neutropenia (LON). They also evaluated the levels of different cytokines (G-CSF, SDF1, BAFF, APRIL) to understand the underlying mechanism of rituximab-induced LON. They observed transient bursts in the blood G-CSF and BAFF concentrations in LON patients, which could partially explain the rituximab-induced LON, as neutrophils are a major source of BAFF and their release is initiated by G-CSF [46,47,48]. However, the complete mechanism of LON following rituximab therapy remains poorly understood. In the majority of cases, neutropenia induced by rituximab therapy resolves spontaneously [49].

Hypogammaglobulinemia is another major concern that has been reported after rituximab therapy [50]. Low levels of immunoglobulins are a significant contributor to infectious complications as they exhibit a prominent role in protective immunity. Tiu et al. evaluated long-term clinical outcomes following rituximab therapy in 142 patients who had autoimmune diseases [51]. Their findings showed a median time of 22.5 months with IgG < 5 g/L in rituximab-treated patients [51]. Casulo et al. observed an association between rituximab administration and an increased risk of symptomatic hypogammaglobulinemia [52]. Almost 39% of their study participants who received multiple rituximab courses had low levels of IgG, whereas 6.6% developed recurrent sinopulmonary infections. However, their conditions improved after intravenous immunoglobulin therapy [52]. A systemic review by Arnold et al. showed that rituximab resulted in serious infections in seven (2.3%) of the total 303 patients, of which four had fatal outcomes [53]. Cohen et al., in their phase 3 trial, evaluated the safety and efficacy of rituximab at twenty-four weeks of assessment. They reported that the rate of serious infection was 5.2/100 patient years following rituximab treatment, compared to 3.7 in the placebo group. The most common infections in the rituximab group included upper respiratory tract infections, sinusitis, nasopharyngitis, urinary tract infections, and bronchitis [11].

A review of the literature by Aksoy et al. investigated 64 cases of rituximab-related viral infections in lymphoma patients. They found that hepatitis B was the most frequent viral infection, followed by cytomegalovirus infection and varicella-zoster virus [12]. Rituximab has also demonstrated higher infection rates in combination therapy either with chemotherapy or immunotherapy. A CLL-10 trial by Eichhorst et al. evaluated two treatment approaches for advanced chronic lymphocytic leukemia (CLL) with fludarabine (F), cyclophosphamide (C), and rituximab (R) (FCR) compared to bendamustine (B) and rituximab (BR). Their findings demonstrated that FCR was superior in terms of efficacy compared to BR; however, FCR was associated with significantly more severe infections compared to the BR group. The risk of infection was higher in participants aged over 65 years, and infections occurred late during treatment, which can be explained by LON [54]. A case report of a 31-year-old woman who received combination therapy with cyclophosphamide and rituximab for indolent lymphoma showed depressed CD4 levels and panhypogammaglobulinema while having recurrent sinus infections [55]. However, the symptoms improved after monthly intravenous immunoglobulin treatments [55].

A phase 3 study evaluated the 6-year outcome in rituximab maintenance treatment for resistant follicular NHL. The findings showed that survival was improved to 74% in the rituximab treatment arm, compared to 64% in the observation arm. However, rituximab maintenance for NHL was significantly associated with grade 3/4 infections (9.7% vs. 2.4%). At a 2-year evaluation, in the observation arm, serum 147 immunoglobulin (Ig) G levels had increased from 6.6 g/L to 7.3 g/L, whereas, in the treatment arm, it was 6.5 g/L at the 2-year assessment and 6.3 g/L at the end of the maintenance therapy [56]. Moulis et al. performed a large population study including patients with immune thrombocytopenia to evaluate the risk of infection after rituximab treatment. Their findings showed that the serious infection rate for the lower respiratory tract was 42.8%, whereas the treatment group had almost a 2.6-times greater risk of developing serious bacterial and viral infections compared to the placebo group [57]. A high prevalence of hepatitis C virus (HCV) infection has been described in B-cell non-Hodgkin’s lymphoma patients. Marignani et al., in a retrospective analysis of 104 consecutive patients, found that nine (8.6%) were HCV-positive, with no reported deaths at the 12-month follow-up [58].

Infections with opportunistic pathogens such as *Pneumocystis jiroveci* after rituximab treatment have been reported in the literature due to impaired cell-mediated immunity. A systemic review of 11 cohort studies showed that lymphoma treated with a rituximab regimen was significantly associated with the risk of pneumocystis pneumonia (PCP) (risk ratio: 3.65) [59]. However, the incidence of PCP was reported to be very low by Barreto et al. in patients with B-cell lymphoma who were treated with rituximab. They analyzed a total of 689 patients at 180 days after the last treatment and found a PCP incidence of 1.51%, which was even below the conventional threshold for the use of prophylaxis [60]. According to the guidelines of the Fifth European Conference on Infections in Leukemia (ECIL-5), trimethoprim/sulfamethoxazole should be given 2–3 times every week for prophylaxis of PCP during at-risk periods after rituximab therapy [61]. The management of CLL has been targeted with anti-CD20 mAbs [62]. Goede et al. compared the efficacy of obinutuzumab and rituximab in combination with chlorambucil in CLL patients. Their findings showed that rituximab addition was associated with grade 3/4 neutropenia (34%) and thrombocytopenia (11%) [63].

Ofatumumab is a fully humanized anti-CD20 monoclonal antibody [64]. After binding to CD20, the Fc portion of ofatumumab induces the cytolysis of B-cells [65]. A phase 3 trial by Byrd et al. evaluated the safety and efficacy of ofatumumab compared to ibrutinib in 391 patients with refractory CLL. Although the incidence of grade 3 or 4 infection was similar in both groups, ofatumumab showed a lower number of infections compared to ibrutinib (54% vs. 70%). Common adverse reactions included rash (8% vs. 4%), pyrexia (24% vs. 15%), and blurred vision (10% vs. 3%) with ibrutinib and ofatumumab, respectively [13]. A phase 3 trial by Davids et al. compared the safety and efficacy of ofatumumab and duvelisib in patients with relapsed/refractory (R/R) CLL/small lymphocytic lymphoma (SLL) [66]. Their findings showed that adverse events of grade 3/4 were more common with ofatumumab compared to duvelisib, including diarrhea (47%/23%), pyrexia (24%/4%), cutaneous reactions (23%/4%), and thrombocytopenia (10%/6%); however, neutropenia was similar in both treatment groups (26%/23%) [66]. Desikan et al. reported that early treatment with ofatumumab in high-risk CLL patients was well tolerated, with only adverse events related to infusion reactions, which were amenable to antihistamine and/or steroid treatment [67].

Obinutuzumab is a humanized monoclonal antibody that was approved in 2017 for the treatment of untreated CLL and untreated or R/R follicular lymphoma (FL) [68,69]. Obinutuzumab leads to the cytolysis of B-cells by activating complement and apoptotic pathways [70]. Marcus et al., in their randomized trial, categorized 1202 patients equally into groups of obinutuzumab-based therapy and rituximab-based therapy for follicular lymphoma [71]. At the 34.5-month follow-up, a higher rate of infection (20%) was reported in the obinutuzumab-treated group, compared to 15.6% in the rituximab group [71]. In a randomized control trial, Goede et al. compared the efficacy of obinutuzumab and rituximab, each combined with chlorambucil, in CLL patients. The overall rate of grade 3/4 infections ranged between 11 and 14% and was not different between groups; however, infusion-related adverse events and neutropenia were more prevalent in the obinutuzumab-treated arm of the study [63].

### 2.2. CD38-Directed Agents and Risk of Infection

The CD38 antigen represents a frequently expressed antigen on plasma cells, which makes them an excellent target for treatment in multiple myeloma (MM) by anti-CD38-directed agents [72]. Daratumumab, an anti-CD38 antibody, has demonstrated efficacy in MM by inducing Fc-mediated cell lysis by cell-mediated toxicity and complement activation [73]. Consequently, daratumumab leads to the depletion of CD38-positive myeloid-derived suppressor cells, T-cells, and B-cells. Several adverse events, including neutropenia, thrombocytopenia, and anemia, have been recorded with the use of daratumumab [74]. Dimopoulos et al. recruited 569 patients with multiple myeloma to investigate the effects of daratumumab and a combination of lenalidomide with dexamethasone. Their findings showed that the severity of daratumumab-treated infections was mild (mostly grade 1 or 2) [74]. Similar findings were shared by Palumbo et al., in their phase 3 trial, who found that most infections were of grade 1 or 2 severity, with only 8.6% exhibiting grade 3 infections [14]. Patients receiving CD38-targeted agents such as daratumumab may be more prone to varicella-zoster virus (VZV) infection [15]. A large phase 3 study was performed by Spencer et al., involving 498 patients with R/R MM [75]. In their CASTOR trial, participants were randomized to receive bortezomib and prednisone or daratumumab, bortezomib, and prednisone. Their findings demonstrated that the daratumumab arm of the study resulted in significantly prolonged neutropenia (12.8% vs. 4.2%) and a greater risk of infectious complications (21.4% vs. 19.0%) [75]. Similarly, Bahlis et al. randomized 569 patients who were previously treated for multiple myeloma into groups receiving daratumumab, dexamethasone, and lenalidomide, or dexamethasone and lenalidomide alone [76]. They reported that the daratumumab group had a higher rate of neutropenia compared to the daratumumab-negative group (5.7% vs. 2.5%) and serious pneumonia (8.1% vs. 8.5%) [76].

### 2.3. CD52-Directed Agents

Alemtuzumab, a humanized monoclonal anti-CD52 antibody that binds to the cell surface CD52 glycopeptide—expressed on almost all human lymphocytes, monocytes, and macrophages—leads to the depletion of CD52-positive B- and T-cells [77]. Alemtuzumab induces antibody-dependent cell-mediated cytolysis, which leads to the depletion of lymphocytes. The low circulating CD4+ lymphocyte count persists for 1 to 2 years after alemtuzumab administration [78,79]. Alemtuzumab is often contraindicated in patients who are affected by human immunodeficiency virus (HIV), primarily due to depleted levels of CD4+ lymphocytes [80]. The immunosuppression caused by alemtuzumab can lead to the reactivation of hepatitis B virus (HBV) infection [81]. Findings of two cases of chronic lymphocytic leukemia with occult HBV infection by Lannitto et al. reported the activation of HBV after immunotherapy with alemtuzumab [82]. Alemtuzumab has also been implicated in herpes infections. Cohen et al., in their phase 3 trial, assessed the comparative effects of alemtuzumab and interferon beta 1a [83]. Patients who were treated with alemtuzumab had higher rates of herpes infections compared to patients treated with interferon beta 1a (16% vs. 2%) [83].

A phase 2 trial by Stilgenbauer et al. reported that alemtuzumab resulted in grade 3/4 neutropenia (56%), thrombocytopenia (57%), and anemia (49%) in CLL patients. Grade 3 to 4 non-cytomegalovirus and cytomegalovirus infections occurred in 29% and 8% of patients, respectively [16]. A smaller study by Poh et al., with only five participants receiving alemtuzumab, reported no cases of CMV infection [84]. CMV reactivation is attributed to the depletion of T-cells following alemtuzumab treatment therapy [85]. A randomized trial by O’Brien et al. evaluated the efficacy of valganciclovir against the reactivation of CMV after alemtuzumab therapy. They showed that none of the patients from the treatment arm showed reactivation, compared to 35% in the group without prophylaxis [86]. Although rare, mycobacterium tuberculosis has been reported in the literature following alemtuzumab therapy. Kim et al. investigated the efficacy of alemtuzumab alone or alemtuzumab-containing therapy. Their findings revealed that out of 182 study participants, 16 were positive for tuberculosis [87]. Other reported infectious complications in their trial included CMV (36%), varicella-zoster virus (13%), and fungal infections (17%) [87]. Bosch et al. reported two cases of patients who received alemtuzumab as part of their renal transplant management and later developed mycobacterium tuberculosis infection [88]. A pooled analysis of 6-year data from the CAMMS223, CARE-MS I, and CARE-MS II studies, and the CAMMS03409 extension study, revealed that the risk of infection with alemtuzumab is mostly mild or moderate, with only 1.0–1.9% serious infections per year. The findings showed that infections decrease over time due to the preservation of protective immunity with time [89].

### 2.4. CD19 Targeted Agents

Anti-CD19 monoclonal antibodies have demonstrated efficacy against several R/R B-cell malignancies [90,91,92,93]. The expression of CD19 is mostly restricted to the B-cell population and it commences in the early developmental stages. Almost all plasma B-cells in peripheral circulation and around 50% of the plasma cells in bone marrow express CD19 on their surfaces. Compared to CD20, it is expressed at an earlier stage [94]. Research has highlighted that infection can occur before and after B-cell-depleting therapies until there is complete recovery of serum immunoglobulins. Inebilizumab is a humanized anti-CD19 mAb that depletes lymphocytes derived from the B-cell lineage [95]. Agius et al. evaluated the safety and tolerability of inebilizumab, an anti-CD19 monoclonal antibody agent. Their findings showed that inebilizumab caused a decrease in immunoglobulin levels and adverse reactions including nasopharyngitis (24%), upper respiratory tract infection (19%), urinary tract infection (14%), urinary tract inflammation (14%), pyrexia (14%), and increased blood pressure (14%). However, most infections were of grade 1 or 2 severity [17]. Recently, two novel anti-CD19 monoclonal antibodies have been approved by the FDA, namely tafasitamab and loncastuximab tesirine, which have been propagated as viable options for the treatment of R/R diffuse large B-cell lymphoma (DLBCL); however, further studies are required to evaluate the infectious complications of these therapies [96].

## 3. Bispecific T-Cell Engagers (BiTE)

Blinatumomab, a bispecific T cell engager (BiTE) antibody, was the first treatment for refractory acute lymphoid leukemia [97]. Blinatumomab crosslinks CD3 on T-cells with CD19 antigen on B-cells, consequently activating T-cell production to eliminate CD19-positive B-cells. As they are cytotoxic to CD19-positive cells due to their cancerous nature, they are likely to cause secondary antibody deficiency [98]. Although the number of T-cells returns to baseline within 7 to 14 days, lower B-cell levels are likely to persist throughout treatment. Consequently, it leads to hypogammaglobinemia, which continues for over a year. Zugmaier et al. reported that the likelihood of serum IgG levels returning to normal is very bleak after blinatumomab treatment [99]. In their phase 2 study, they demonstrated that upon follow-up for 255 to 1605 days (median, 457.5 days) among six patients treated with blinatumomab, five out of six patients did not recover their IgG levels. Only one subject was able to obtain normalcy after 2 years. As CD19 is expressed on plasma blasts, their targeted therapy induced more profound immune suppression. They demonstrated that grade 3 infections were reported by 9.5% of participants [99].

A phase 3 RCT by Kantarjian et al. reported that almost 6% of participants reported depleted IgG levels when treated with blinatumomab, compared to 0.9% in the chemotherapy arm of the study [100]. However, these results were not translated to neutropenia outcomes, as blinatumomab caused lower neutropenia (38%), compared to 58% in the chemotherapy group, whereas AEs of grade 3 or more were also less frequent, at 87% in the blinatumomab group compared to 92% in the chemotherapy arm of the study [100]. Further characterization of blinatumomab-related infections was provided by Topp et al., in their phase II clinical trial [91]. They reported grade 3 infections in participants, with a higher number of catheter-related infections (9.5%), followed by bacterial/*Escherichia* sepsis (4.8%) and bronchopneumonia (4.8%) [91]. Similar observations were shared by another phase 2 trial in patients aged over 65 years with relapsed/refractory B-precursor acute lymphoblastic leukemia (r/r ALL). The authors reported that grade 3 or more AEs were reported in 86% of participants, whereas infections were seen in 39% of patients [18].

## 4. Bruton’s Tyrosine Kinase (BTK) Inhibitors

The management of hematological disorders has undergone profound changes in recent years with the rise of novel anti-cancerous agents [101]. Several Bruton tyrosine kinase (BTK) inhibitors, including ibrutinib, acalabrutinib, and zanubrutinib, have emerged that inhibit Bruton tyrosine kinase (BTK). Ibrutinib, a first-in-class BTK drug, has been attributed to an increased population of activated T-cells and diminished Treg/CD4+ T-cell ratios while imparting its immunomodulatory effects against CLL through the inhibition of BTK and IL-2-inducible T-cell kinase (ITK) [102]. However, ibrutinib use has shown several adverse reactions, such as diarrhea, upper respiratory tract infection, hyperuricemia, pyrexia, pneumonia, musculoskeletal pain, and atrial fibrillation. Severe infections of grade 3 or higher have been reported in 35% of patients. The most commonly cited hematological AEs include thrombocytopenia, neutropenia, and anemia [19]. Acalabrutinib is a novel BTK inhibitor that is recommended for the treatment of CLL. The efficacy of acalabrutinib is well established, with recent research demonstrating its better safety profile. A meta-analysis of three RCTs with 1362 patients reported a significantly lower relative risk of infection in acalabrutinib-treated patients compared to non-acalabrutinib-based therapies [103]. Another BTK inhibitor, zanubrutinib, has demonstrated a better safety profile compared to other targeted agents. Investigations by Trotman et al. in 73 Waldenström macroglobulinemia patients concluded that long-term treatment with single-agent zanubrutinib demonstrated a durable response with an acceptable safety profile [104]. AEs mostly included grade 3 diarrhea, neutropenia, and atrial fibrillation [104]. A review by Tillman et al. reported that infectious complications such as pneumonia developed in 56% of patients taking single-agent ibrutinib and 52% of those on combination therapy [105].

## 5. Phosphoinositide 3-Kinase (PI3K) Inhibitors

The activation of receptors on B-cells leads to downstream signaling pathways that ensure proliferation, cell survival, and motility. Normally, these pathways include phosphatidylinositol 3-kinase (PI3K), protein kinase B (AKT), and mammalian target of rapamycin (mTOR) and are often activated in B-cell malignancies [106]. The activation of PI3K has been implicated in the recruitment of several intracellular enzymes, leading to cancerous cell proliferation [107]. Therefore, PI3K represents an important target for anticancer therapy in several hematological malignancies [108]. Idelalisib is an orally bioavailable, small-molecule, reversible inhibitor of PI3K-δ [109]. It was the first PI3K inhibitor that was approved for the treatment of CLL and follicular lymphoma (FL) [110]. Other PI3K inhibitors including duvelisib and copanlisib were later approved. A phase 3 trial by Furman et al. evaluated idelalisib along with rituximab in the treatment of relapsed CLL. They reported that serious adverse events occurred in 40% of patients, including pneumonia, pyrexia, and febrile neutropenia [111].

A phase 3 trial by Zelenetz et al. compared the addition of idelalisib or a placebo to bendamustine and rituximab in patients with relapsed or refractory CLL. Their findings showed that 60% of the patients in the idelalisib arm developed neutropenia, whereas 23% demonstrated febrile neutropenia [112]. They also observed a higher frequency of infections in the idelalisib-treated group compared to the placebo group (69% vs. 59%). Pneumonia of bacterial origin was reported in 14% of patients in the idelalisib arm of the study and CMV infection (6%), PJP (2%), and pulmonary mycoses were also observed in the treated group [112]. Similar results were shared by Jones et al., who reported grade 3 or higher neutropenia and pneumonia in 34% and 14% of patients, respectively, with idelalisib treatment, compared to 16% and 8% in ofatumumab monotherapy [113]. Lymphocytosis is often reported in patients who receive PI3K inhibitors as a monotherapy [111,114,115]. The SEIFEM retrospective study reported infectious complications with ibrutinib and idelalisib in lymphoproliferative disorders [20]. Almost 32.1% (36/112) of patients experienced one or more infections. Viral infections/reactivations were observed in 61.5% (16/26) of patients, with a major share of CMV infection [20]. PI3K inhibitors have demonstrated a variable risk of infection, with some depicting an acceptable risk of infection and others culminating in the termination of the trial owing to severe adverse reactions [116,117].

## 6. Janus-Associated Kinase (JAK) Inhibitors

Janus-associated kinases (JAKs) are a family of four receptors that mediate the signaling of cytokine receptors via the signal transducer and activator of the transcription (STAT) pathway. They are involved in the proliferation of a variety of cells but play a crucial role in immune and hematopoietic cells [118]. Ruxolitinib, an inhibitor of JAK1 and JAK2, was approved in 2011 for the treatment of myelofibrosis. It leads to the downregulation of T-helper-cell type 1 (Th1) responses and cytokines including IL-1, IL6, and TNFα [119]. A phase 3 randomized trial by Vannucchi et al. investigated ruxolitinib versus standard therapy for polycythemia vera. Their findings showed that grade 3/4 anemia and thrombocytopenia occurred in 2% and 5% of participants, respectively, whereas the corresponding percentages were 0% and 4% in standard therapy. Herpes zoster infection was much higher (6%) in the ruxolitinib-treated group, compared to 0% in the standard therapy group [21]. Similar observations were shared by Verstovsek et al. in patients with myelofibrosis who underwent ruxolitinib treatment. They found that herpes zoster infections were more common in the ruxolitinib-treated group compared to the untreated group; however, other infectious complications were similar in both groups [120]. Similarly, a review and meta-analysis by Lussana et al. reported that ruxolitinib treatment was associated with a higher risk of herpes zoster infection compared to the control group [121].

## 7. B-Cell Lymphoma 2 (BCL-2) Inhibitors

BCL-2 inhibitors represent a class of anti-tumor agents that are selective inhibitors of the anti-apoptotic protein B-cell lymphoma 2 (Bcl-2). Upon binding to Bcl-2, they inhibit its activity, which restores apoptotic processes in tumor cells [122,123]. Venetoclax has shown efficacy in the treatment of relapsed chronic lymphocytic leukemia. However, its use has been associated with an increased risk of infection, mainly due to neutropenia. A clinical trial by Davids et al. observed a higher incidence of grade 3/4 neutropenia, which resulted in infections in almost 15% of patients [124]. The safety analysis of 350 CLL patients showed that infection of any type was observed in 72% of patients, with a major share of respiratory infections and fever [124]. These findings were supported by DiNardo et al., who reported severe adverse events including sepsis, bacteremia, lung infection, and respiratory problems within 30 days of the first venetoclax treatment [22]. A study by Lee et al. investigated the risk of infection in 122 AML patients treated with venetoclax. Their findings showed that 18% were diagnosed with *Aspergillus* infections [125]. Similarly, a study by DiNardo et al. investigated the impact of venetolax treatment in 43 patients (AML (91%), MDS (5%), or BPDCN (5%)). Their findings showed that 72% patients developed grade ≥ 3 infections. The most common infections included pneumonia (17.40%), bloodstream infections due to Gram-negative (30%) or Gram-positive (23%) bacteria, cellulitis (21%), and invasive fungal infections (19%) [126] (Table 2).

## 8. Other Novel Agents

As the therapeutic landscape of hematological malignancies is being enriched with novel targeted agents, there are several targeted agents are still in the process of safety evaluations. Due to a lack of substantial evidence, such agents are only summarized here. For example, brentuximab vedotin is a conjugated antibody directed against CD30 that was approved in 2011 for the treatment of Hodgkin’s lymphoma, R/R anaplastic lymphoma, and cutaneous T-cell lymphoma [130]. The major risk factor of infectious complications arises due to the tendency of this drug to cause neutropenia [127,128]. Tudesq et al. reported cytomegalovirus infection after brentuximab vedotin treatment [131]. Inotuzumab ozogamicin is a CD22-directed antineoplastic agent that is used in the treatment of B-ALL [129,132]. Kantarjian et al. reported lower rates of neutropenia compared to standard therapy; however, veno-occlusive liver disease was observed in 11% (15/109) who received inotuzumab ozogamicin and in 1% in standard therapy [129]. FMS-like tyrosine kinase 3 (FLT3) inhibitors are novel agents that target FLT3, a receptor tyrosine kinase that is expressed primarily in the hematopoietic compartment [133]. Over 30–35% of patients suffering from acute myeloid leukemia express mutations of FLT3-ITD and FLT3-TKD, consequently resulting in the prolonged activation of proteins that promote cell proliferation and survival [134]. A review by Xu et al. demonstrated that FLT3 inhibitors improved outcomes in the induction/reinduction stage of FLT3(+) AML; however, adverse reactions including thrombocytopenia, neutropenia, anemia, cardiac abnormalities, dyspnea, and cough were observed [135]. The breakpoint cluster region/Abelson leukemia virus (BCR-ABL) inhibitors have been used to treat CML, ALL, and other hematological malignancies. Imatinib was the first approved drug in this class for the treatment of CML or ALL [136]. Kalmanti et al. evaluated the 10-year safety and efficacy of imatinib in CML. Their findings showed that the eight-year probability of grade 3/4 adverse events was 22% [137]. IDH inhibitors are another type of targeted therapy that target genetic mutations in the isocitrate dehydrogenase genes (IDH1 and IDH2) in acute myeloid leukemia (AML), occurring in up to 30% of AML cases [138]. Most infectious complications with these agents are mild in nature [139].

Immune checkpoint inhibitor therapies, specifically targeting programmed death 1 (PD-1) and cytotoxic T-lymphocyte-associated protein 4 (CTLA-4), have brought significant advancements in the treatment of hematological malignancies. The effectiveness of combining nivolumab, an anti-PD-1 antibody, with ibrutinib, a Bruton’s tyrosine kinase inhibitor, was assessed in patients with r/r NHL by Younes et al. [140]. One notable adverse event, which affected dosage limitations, was grade 3 hyperbilirubinemia. Neutropenia (grade 3–4) was the most frequently reported severe adverse event, occurring in 28% of patients, followed by anemia in 23% of patients [140]. To date, the FDA has granted approval to two chimeric antigen receptor (CAR) T-cell therapies targeting CD19. These therapies, namely axicabtagene ciloleucel (axi-cel) and tisagenlecleucel, are available for use in patients with rel/ref large B-cell lymphoma who have undergone at least two prior treatment regimens. Tisagenlecleucel received FDA approval in August 2017 for the treatment of B-cell acute lymphoblastic leukemia, based on findings from a small phase II study conducted by Maude et al. This study reported that 73% of patients experienced grade 3 or 4 adverse events [141]. Another study conducted by Abramson et al. evaluated Lisocabtagene maraleucel (liso-cel). The most frequently observed grade 3 or worse adverse events in this study were neutropenia in 60% patients, anemia in 37%, and thrombocytopenia in 27%. Cytokine release syndrome and neurological events were observed in 42% and 30% patients, respectively. Furthermore, grade 3 or worse cytokine release syndrome and neurological events were seen in 2% and 10% patients, respectively [142]. Hematologic malignancies pose a challenge for curative treatment due to the presence of clonal heterogeneity and the emergence of drug resistance. Tumor vaccines have been investigated as a therapeutic approach to stimulate the host immune system. A study conducted by Frank et al. examined the effectiveness of tumor-specific idiotype vaccines in patients with B-cell lymphoma [143]. Their findings revealed that the administration of these vaccines resulted in certain side effects, including local skin reactions such as erythema, tenderness, and induration, as well as myalgias/arthralgias and fever. Another study by Stephen et al. explored the safety and efficacy of vaccination with patient-specific tumor-derived antigens in individuals experiencing their first remission from follicular lymphoma. Grade 1 to 2 AEs, particularly injection-site reactions with erythema and induration lasting for a few days, were common in both the treatment and control groups. However, severe grade 3 to 4 AEs were rare, and there were no deaths related to the administration of the idiotype vaccine [144].

## 9. Impact of Targeted Therapies on SARS-CoV-2 Infections

The intersection of coronavirus disease 2019 (COVID-19) infection and targeted therapies poses a complex therapeutic dilemma for healthcare providers as both cause significant morbidity and mortality. The interplay between the host immune system, underlying hematological malignancy, and targeted therapies can have significant impacts on the course of COVID-19 illness. Patients suffering from CLL have demonstrated augmented vulnerability to the severe manifestation of the novel coronavirus (COVID-19), irrespective of their disease phase or their current treatment regimen [145,146]. A joint retrospective international multicenter study by ERIC, the European Research Initiative on CLL, and CLL Campus evaluated 190 patients with confirmed CLL and COVID-19 [146]. The majority of participants (79%) presented with severe COVID-19 (need for oxygen and/or intensive care admission). The rate of hospitalization was significantly lower in ibrutinib-treated patients (*p*-value < 0.05) compared to patients on alternative regimens [146]. However, these findings were not supported by Courtre et al., who found no improvement with the addition of ibrutinib in the routine standard of care [147]. Some studies have demonstrated impaired serologic responses following COVID-19 vaccination in CLL patients undergoing targeted therapies, particularly anti-CD20 antibody therapy [148,149]. Herishanu et al. investigated the antibody response following the third dose of the BNT162b2 mRNA vaccine in CLL/SLL patients who failed to achieve a humoral response after standard two-dose vaccination [150]. Antibodies against severe acute respiratory syndrome coronavirus 2 (SARS-CoV-2) were measured 3 weeks post-vaccination. Their findings revealed that 23.8% of the 172 CLL patients had an antibody response. The response rate was lower among patients who were actively treated (12.0%) compared to those who were treatment-naïve (40.0%) and off-therapy (40.6%). Furthermore, the lowest response rate was observed in patients receiving Bruton’s tyrosine kinase inhibitors or venetoclax with or without anti-CD20 antibody treatment (15.3% and 7.7%, respectively). Only a limited proportion of patients treated with anti-CD20 antibodies less than 12 months prior to vaccination (3.6%) demonstrated an antibody response [150]. Similarly, Parry et al. investigated spike-specific antibody responses following COVID-19 vaccination in 299 CLL patients and healthy subjects [149]. Their results showed that 34% of CLL patients demonstrated spike-specific antibody responses, which was significantly lower compared to 94% of healthy participants, with 104-fold lower antibody titers in the CLL group. After the second vaccine, the response rate increased to 75% in CLL patients but was lower than that of the control group (100%) [149]. A study by Shen et al. assessed the immune response in 181 CLL and monoclonal B-cell lymphocytosis (MBL) patients in correlation with their seroconversion status following the administration of two doses of the SARS-CoV-2 spike protein IgG assay [148]. The results revealed that 79.2% of CLL patients and 50% of MBL patients failed to achieve seroconversion after the first dose, whereas 45% of CLL and 9.5% of MBL patients remained seronegative following the second dose. Univariate analysis indicated a significant correlation between the antibody level after dose two and the pre-vaccination levels of reduced IgM (*p* < 0.0001), IgG2 (*p* < 0.0351), and IgG3 (*p* < 0.0457), as well as the therapy received by the CLL patient within the previous 12 months (*p* < 0.001) [148]. Blixt et al. evaluated sixty consecutive CLL patients during the first 13 months of the pandemic. Seroconversion to anti-SARS-CoV-2 antibodies was observed in 82% of the 40 tested patients, with 17/22 and 8/11 patients testing positive for antibodies at 6 and 12 months, respectively [151]. The risk of COVID-19 in patients undergoing targeted therapies is an intriguing topic; however, we do not present a detailed discussion here as this is beyond the scope of our review. As significant evidence has emerged in this regard, a separate review on this aspect would be more suitable.

## 10. Conclusions

The advent of novel targeted therapies has broadened the treatment horizons of hematological malignancies. This everchanging treatment landscape has brought several challenges to practicing clinicians, who require updated knowledge to tackle the broad range of clinical manifestations arising following targeted therapies. Patients who receive targeted therapies for hematological malignancies are prone to infectious complications as some of these therapies have a profound impact on the immune status of the individual. The risk of infection can vary among individuals owing to the underlying malignancy and previous therapeutic treatments. This highlights the need for a thorough compilation of knowledge on this subject, collating the clinical evidence of infectious complications in several targeted agents. However, several challenges emerge in this setting as most targeted therapies are used in combination with chemotherapy or other immunosuppressive agents such as glucocorticoids. This further exacerbates the challenge to identify the attributable infection risk of one particular agent. The infectious consequences of targeted therapies can be better managed by adopting screening of latent infection and management practices for hematological malignancies.

## Figures and Tables

**Table 1:** List of targeted therapies in hematological malignancies and their risks of infection.

Therapeutic Intervention	Drugs	Mode of Action	Approved Indication	Risk of Infection
CD20-targeted therapy	Rituximab, obinutuzumab, ofatumumab	Complement-dependent cytotoxicity, antibody-dependent cell-mediated cytotoxicity	Non-Hodgkin’s lymphoma (NHL) and chronic lymphocytic leukemia (CLL)	Rituximab treatment can result in severe infections, including upper respiratory tract infections, sinusitis, nasopharyngitis, urinary tract infections, and bronchitis [11,12]. Viral infection with hepatitis B, cytomegalovirus infection, and the varicella-zoster virus has been observed in rituximab-treated patients [13].
CD38-targeted therapy	Daratumumab	Complement-dependent cytotoxicity, antibody-dependent cell-mediated cytotoxicity	Multiple myeloma	Most infections with daratumumab are of mild severity (grade 1 or 2) [14]. Patients undergoing daratumumab therapy are prone to varicella-zoster virus (VZV) infection [15].
CD52-targeted therapy	Alemtuzumab	Complement-dependent cytolysis (CDC) and antibody-dependent cellular cytotoxicity	Chronic lymphatic leukemia	Alemtuzumab leads to grade 3/4 neutropenia, thrombocytopenia, non-cytomegalovirus, cytomegalovirus infections, and anemia in chronic lymphocytic leukemia (CLL) patients [16].
CD19-targeted therapy	Inebilizumab	Modulates B-cell receptor (BCR)-dependent and independent signaling pathways	Acute lymphocytic leukemia	Inebilizumab-related infections include nasopharyngitis, upper respiratory tract infection, urinary tract infections, and hypertension [17].
Bispecific T-cell engagers (BiTE)	Blinatumomab	It crosslinks CD3 on T-cells with CD19 antigen on B-cells, consequently resulting in the activation of T-cells and proliferation of cytolytic proteins to eliminate CD19-positive B-cells	Refractory acute lymphoid leukemia	The likelihood of serum IgG levels returning to normal is very bleak after blinatumomab treatment [18].
Kinase inhibitors	Ibrutinib, acalabrutinib, zanubrutinib	Inhibit Bruton tyrosine kinase (BTK)	Mantle cell lymphoma, chronic lymphocytic leukemia, Waldenström macroglobulinemia	Ibrutinib use is correlated with various infections, such as diarrhea, upper respiratory tract infections, pyrexia, pneumonia, musculoskeletal pain, and atrial fibrillation. Hematological AEs include thrombocytopenia, neutropenia, and anemia [19].
Phosphoinositide 3-kinase (PI3K) inhibitors	Idelalisib, duvelisib, copanlisib	Inhibition of PI3K pathways	Chronic lymphocytic leukemia (CLL) and follicular lymphoma (FL)	Following idelalisisb, almost 32.1% (36/112) of patients experienced one or more infections. Viral infections/reactivations were observed in 61.5% (16/26) of patients with a major share of CMV infection [20].
Janus-associated kinase (JAK) inhibitors	Ruxolitinib	Inhibitor of Janus-associated kinases (JAKs)	Polycythemia vera	Ruxolitinib treatment was associated with grade 3/4 anemia and thrombocytopenia in 2% and 5% of participants, respectively, whereas corresponding percentages were 0% and 4% in standard therapy [21].
BCL-2	Venetoclax	Selective inhibitors of the anti-apoptotic protein B-cell lymphoma 2 (Bcl-2)	Chronic lymphocytic leukemia, acute myeloid leukemia	Venetoclax use has been associated with severe adverse events including sepsis, bacteremia, lung infection, and respiratory problems, which are observed within 30 days of the first venetoclax treatment [22].

**Table 2:** Summary of grade 3/4 infection and neutropenia incidence in hematological patients treated with targeted therapies.

Class of Therapy	Agent	Severe Infection (Grade 3/4)	Neutropenia	Immune Response	Reference
Anti-CD20 monoclonal antibodies	Rituximab	5.2% [11]	Late onset of neutropenia	Hypogammaglobulinemia	[45,50]
	Ofatumumab	54%	Yes	Present	[13,66]
	Obinutuzumab	11 to 14%	Yes	Present	[63]
CD38-directed agent	Daratumumab	8% (grade 3 only)	Prolonged neutropenia	NK cell activation and degranulation	[14]
CD52-directed agent	Alemtuzumab	29% (grade 3), 8% (grade 4)	Present	Early hyper-repopulation of B-cells	[16]
CD19-targeted agent	Inebilizumab	Mostly grade 1 or 2	Rare	Selective depletion of CD19-positive B-cells	[17]
Bispecific T-cell engagers (BiTE)	Blinatumomab	9.5%	Present	Hypogammaglobinemia for over a year	[99]
Bruton’s tyrosine kinase (BTK) inhibitors	Ibrutinib	35%	Present	Prolonged treatment with ibrutinib restores humoral immunity	[19]
	Zanubrutinib	58.4%	Present	Hampers the cytotoxic activity of NK cells	[104]
Phosphoinositide 3-kinase (PI3K) inhibitors	Idelalisib	34%	Febrile neutropenia	Impairs cell adhesion, leading to transient lymphocytosis	[113]
Janus-associated kinase (JAK) inhibitors	Ruxolitinib	2–5%	Present	Alterations of DC function	[21]
B-cell lymphoma 2 (BCL-2) inhibitors	Venetoclax	15%	Present	Rapid apoptosis of CLL cells	[124]
CD30 directed agents	Brentuximab, vedotin	39%	Neutropenia	BV-induced peripheral neurotoxicity	[127,128]
CD22-directed agents	Inotuzumab, ozogamicin	11%	Present	Unclear	[129]

## Data Availability

Not applicable.
